# Prevalence and Risk Factors for Peripheral Neuropathy in Chinese Patients With Gout

**DOI:** 10.3389/fneur.2022.789631

**Published:** 2022-02-24

**Authors:** Kaifeng Guo, Nan Liang, Mian Wu, Lihui Chen, Haibing Chen

**Affiliations:** ^1^Department of Endocrinology and Metabolism, Shanghai 10th People's Hospital, School of Medicine, Tongji University, Shanghai, China; ^2^Department of Endocrinology and Metabolism, Minhang Hospital, Fudan University, Shanghai, China; ^3^Department of Endocrinology and Metabolism, Shanghai Jiaotong University Affiliated Sixth People's Hospital, Shanghai, China; ^4^Department of Endocrinology and Metabolism, The Affiliated Suzhou Hospital of Nanjing Medical University, Suzhou Municipal Hospital, Suzhou, China

**Keywords:** gout, peripheral neuropathy, vibration perception threshold, tophi, prevalence

## Abstract

**Objective:**

Peripheral neuropathies (PNs) are a group of disorders that affect the peripheral nervous system. PN in gout has been scarcely described. This study is conducted to determine the prevalence and related risk factors of PN, as assessed by vibration perception threshold (VPT) in patients with gout.

**Methods:**

A total of 442 patients were included in the cross-sectional study. The VPT values were measured by using the Biothesiometer sensory quantitative tester on each patient. The VPT value of either limb higher than 15 V was considered abnormal and is considered to have PN. The univariate and multivariate logistic regression models were used to identify risk factors for PN in patients with gout.

**Results:**

We included 442 patients with gout, 97.5% men, 26.9% tophaceous gout, mean age 45.5 ± 15.2 years, and 7.4 ± 4.6 years of disease duration. The prevalence of PN in patients with gout was 11.1%. Patients in the abnormal VPT group were older, had a longer gout duration, and had significantly higher levels of waist circumference, waist-to-hip ratio, systolic blood pressure (SBP), and erythrocyte sedimentation rate (ESR), as compared to patients in the normal vibration group (*P* < 0.05). The univariate logistic regression analysis demonstrated that there was a significant association between abnormal VPT and age, duration of gout, SBP, C-reactive protein, ESR, presence of tophi, and estimated glomerular filtration rate (eGFR) in all the subjects (*P* < 0.05). The multivariate logistic regression analysis indicated that age (odds ratio, 1.094) and presence of tophi (odds ratio, 1.048) were independent risk factors for PN in patients with gout.

**Conclusion:**

The abnormal VPT was significantly correlated with age and presence of tophi in patients with gout and the VPT level may be useful as a screening tool for assessment of PN in gout.

## Introduction

Gout is the most prevalent inflammatory arthritis in the world. A meta-analysis of 30 studies published from 2000 to 2016 found a pooled prevalence of gout in the adult population in China of 1.1% (1). Gout is associated with multiple comorbidities, such as hypertension (2), kidney disease (3), neurodegenerative diseases (4), and cardiovascular disease (5). A thorough understanding of the complications of gout and early prevention and treatment are very important to improve the quality of life of patients.

Peripheral neuropathies (PNs) are disorders of the peripheral nervous system that affect the myelin sheath, anterior horn cells, axons, and/or sensory neurons (6). There is an increasing prevalence of PNs worldwide. PN is more common in patients with diabetes mellitus, vitamin deficiencies, amyloidosis, alcohol abuse, uremia, infectious and autoimmune diseases, and exposure to environmental toxins. Among them, diabetic PN (DPN) has become the most common cause of PN. Recently, several observational studies suggest that hyperuricemia is also associated with PNs including chronic inflammatory demyelinating polyneuropathy (CIDP) (7), diabetic sensorimotor polyneuropathy (8), and also in healthy subjects (9). However, to the best of our knowledge, epidemiological data regarding the prevalence of PN in patients with gout have been scarcely described. There are several reports of compression neuropathy, especially carpal tunnel neuropathy, due to hyperuricemia (gout) in which tophaceous deposits were found at surgery.

Early detection and good urate control may delay or prevent adverse outcomes resulting from PN, thereby improving quality of life of patients. As so far, there was no clear diagnostic criterion for PN in patients with gout, which is mainly a diagnosis of exclusion. Quantitative sensory testing (QST) that focuses on vibration perception threshold (VPT) potentially offers a quick, accurate, and inexpensive screening instrument to evaluate high-risk patients in the clinic. Several researchers agreed that the diagnosis of peripheral sensory neuropathy can be confirmed with VPT (10, 11). Over years of having gout, patients with gout will have a loss of sensation of vibration, leading to the VPT that is greater than a healthy patient.

Therefore, our main objective was to explore the factors associated with VPT, to determine whether hyperuricemia is related to abnormal VPT, as an indicator of large nerve fiber dysfunction, in patients with gout, and to estimate the prevalence and risk factors associated with PN in a large sample of Shanghai Chinese patients with gout, which may lead to improved preventive measures and care for gout.

## Methods

### Study Population

A total of 442 patients (431 men and 11 women) were included in the cross-sectional study. All the subjects were recruited from the Department of Endocrinology and Metabolism, Shanghai Jiaotong University Affiliated Sixth People's Hospital between the years 2015 and 2020. The diagnosis of gout was performed according to the 2015 Gout Classification Criteria. All the patients did not receive regular urate-lowering treatment. Patients with the following conditions were excluded: (1) patients for whom VPT data was not available, (2) subjects who had neuropathy resulting from other causes, such as carpal tunnel syndrome (CTS), toxic peripheral neuritis, infectious polyneuritis, cerebral infarction, and Guillain–Barré syndrome (GBS), and (3) patients with diabetes mellitus. All the patients underwent an interview and provided a history of alcohol consumption (non-drinker and current) and smoking habits. This study was performed according to the principles of the Declaration of Helsinki and was approved by the Ethics Committee of Shanghai Jiaotong University Affiliated Sixth People's Hospital. All the study subjects provided informed consent.

### Anthropometric and Biochemical Measurements

Demographic and clinical data, which included waist circumference, hip circumference, weight, height, age, sex, gout duration, and blood pressure, were recorded. Body mass index (BMI) was calculated as weight in kilograms divided by squared height in meters (kg/m^2^). Blood pressure was measured by using a standard mercury sphygmomanometer after the subject had been seated for at least 30 min. The waist-to-hip ratio (WHR) was calculated by dividing waist by hip circumference (cm). Venous blood samples were collected between 08:00 and 09:00 h after a 12-h fast. Serum urea nitrogen, creatinine, urate, alanine aminotransferase (ALT), aspartate aminotransferase (AST), and lipid profiles, including measurements of total cholesterol (TC), triglycerides (TGs), high-density lipoprotein cholesterol (HDL-C), and low-density lipoprotein cholesterol (LDL-C), were measured on the Hitachi 7600 Analyzer by using an enzymatic assay (Hitachi Incorporation, Tokyo, Japan). Serum C-reactive protein (CRP) was measured by a particle-enhanced immunoturbidimetric assay (Dade Behring Incorporation, Newark, Delaware, USA). Estimated glomerular filtration rate (eGFR) was estimated by using the Modification of Diet in Renal Disease (MDRD) Study formula as follows: 186 × [serum creatinine (mg/dl)]^−1.154^ × (age)^−0.203^ × (0.742 if female) (12). The diagnosis of hypertension and hyperlipidemia in this study has been previously described in detail (13).

### Vibration Perception Threshold Measurements

All the VPT values are completed by the same operator by using the Biothesiometer sensory quantitative tester. The operation procedure adopts the method of the International Working Group on the Diabetic Foot (14): the examinee is lying down, eyes closed, and undergoing examination in a quiet and relaxing environment. Turn on the power of the instrument and touch the vibrating head to the bone protrusions at the base of the thumbs of patient under a low voltage of 5 V and then gradually increase the current intensity and the amplitude of the vibrating button until it can be sensed by the examinee. Take the voltage value at this time. Repeat the above operation twice in succession and take the average value thrice as the VPT value of this side. Patients were divided into two groups by vibration perception: the normal vibration group with the VPT value <15 V for both feet and the abnormal VPT group with a vibration perception of ≥15 V for at least one foot (15). The greater VPT value of the bilateral limb was used in the analysis.

### Ultrasonographic Assessments

All the subjects underwent ultrasound and the diagnosis of tophi was determined as previously described (16). Briefly, ultrasound examinations were performed by a single experienced ultrasonographer blinded to the clinical information of patients including gout status and serum urate. The diagnosis of tophi was determined based on the ultrasound results showing that there is a hypoechoic to hyperechoic inhomogeneous material, often surrounded by a small anechoic rim.

### Statistical Analysis

All the statistical analyses were performed by SPSS version 17.0 software (IBM Incorporation, Chicago, Illinois, USA). For normally distributed variables, the data are expressed as mean ± SD and nonnormally distributed variables were presented as medians (quartiles 25 and 75%). Student's unpaired *t*-tests were used to compare the two groups. We performed the univariate and multivariate logistic regression analyses to identify variables associated with PN (VPT > 15 V) in patients with gout. In all the statistical tests, a value of *P* < 0.05 was considered significant.

## Results

### Baseline Characteristics of the Study Population

**Figure 1:**
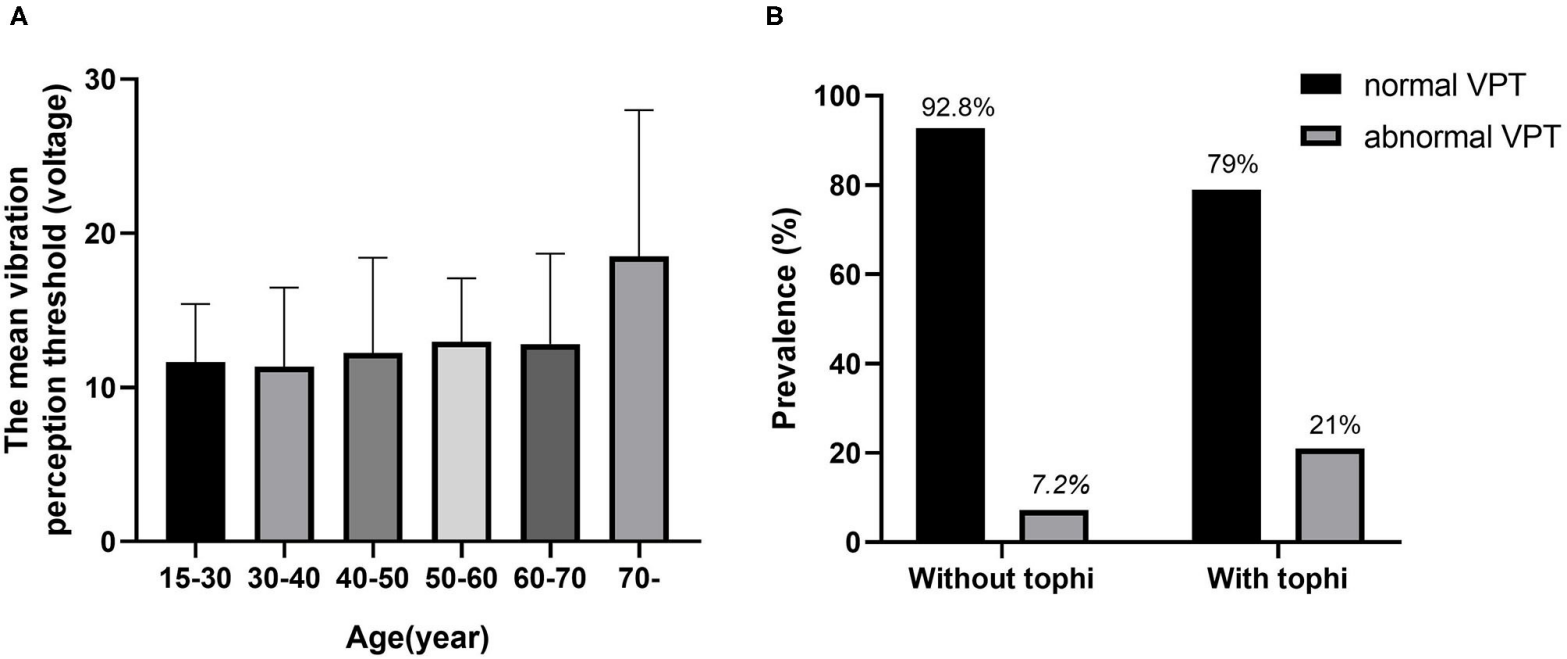
The relationship between vibration perception threshold (VPT) and age and tophi. **(A)** The mean VPT levels according to the age categories. **(B)** Comparison of the prevalence of normal VPT and abnormal VPT between gout patients with and without tophi.

In total, 442 adult patients with gout (431 men and 11 women) with available data were included in the analysis. The clinical characteristics of the participants are shown in Table 1. The mean age of the participants was 45.49 ± 15.16 years and the mean duration of gout was 7.4 ± 4.6 years. Patients were divided into the two groups by vibration perception; the prevalence of PN (VPT > 15 V) in patients with gout was 11.1%. Patients in the abnormal VPT group were older (61.61 ± 14.47 vs. 43.46 ± 14.01 years), had a longer gout duration (11.36 ± 8.09 vs. 7.04 ± 6.51 years), and had significantly higher levels of waist circumference, WHR, systolic blood pressure (SBP), and erythrocyte sedimentation rate (ESR), as compared to patients in the normal vibration group (*P* < 0.05). Meanwhile, no statistical difference was observed in the sex, BMI, drinker, and smoking history levels between the groups. The prevalence of the abnormal VPT group was higher in progressively increasing age categories (see Figure 1A) and was also markedly higher in patients with gout with tophi than in those without tophi (see Figure 1B). In this study, the prevalence of tophi in patients with gout was 26.92%.

**Table 1:** Clinical characteristics of patients with gout according to the vibration perception threshold (VPT) (voltage) stages (*n* = 442).

**Variables**	**VPT <15 V**	**VPT > 15 V**	***P***
*n* (%)	393 (88.9%)	49 (11.1%)	
Male gender, *n* (%)	385 (98.0%)	46 (93.9%)	0.213
Age (years)	43.46 ± 14.01	61.61 ± 14.47	<0.001
Smoker (%)	25.4%	28.6%	0.214
Drinker (%)	19.8%	20.4%	0.975
BMI (kg/m^2^)	26.38 ±7.24	26.22 ± 4.69	0.885
Height (cm)	171.8 ± 10.3	171.4 ± 7.0	0.753
Waist circumference (cm)	93.46 ± 10.23	96.04 ± 6.17	0.016
Waist/hip ratio	0.93 ± 0.07	0.95 ± 0.06	0.032
Duration of gout (years)	7.04 ± 6.51	11.36 ± 8.09	0.001
Hypertension (%)	31.6%	63.0%	<0.001
Hyperlipidaemia (%)	36.4%	30.6%	0.425
SBP (mmHg)	128.64 ± 15.65	137.8 ± 15.73	<0.001
DBP (mmHg)	83.2 ± 11.24	85.4 ± 10.84	0.208
HbA1c (%)	5.60 ± 0.49	5.70 ± 0.42	0.197
FPG (mmol/L)	5.46 ± 0.65	5.51 ± 0.53	0.671
TC (mmol/L)	5.06 ± 0.99	4.89 ± 0.93	0.334
TG (mmol/L)	2.08 (1.47–3.16)	1.82 (1.46–2.58)	0.322
LDL-c (mmol/L)	3.05 ± 0.86	3.02 ± 0.84	0.818
HDL-c (mmol/L)	1.04 ± 0.23	1.06 ± 0.29	0.608
Serum creatinine (μmol/L)	83.0 (67.0–95.0)	95.0 (71.2–108.0)	0.007
eGFR (ml/min/1.73 m^2^)	93.08 ± 22.08	76.83 ± 21.26	<0.001
Urate (μmol/L)	524.0 (457.0–588.5)	602.0 (505.0–685.6)	0.591
ALT (U/L)	28.0 (18.5–42.5)	20.0 (16.0–33.0)	0.416
AST (U/L)	22.0 (18.0–28.0)	21.0 (17.0–30.0)	0.622
GGT (U/L)	35.0 (24.0–57.0)	31.0(23.0–42.0)	0.053
CRP (mg/L)	1.31 (0.67–3.30)	2.26 (0.87–5.11)	0.152
ESR (mm/h)	12.0 (4.3–21.0)	25.0 (9.0–40.0)	0.031
Anti-hypertensive drug users (%)	20.3%	42.9%	<0.001
Colchicine users (%)	47.3%	59.2%	0.117
Presence of tophi (%)	24.8%	53.2%	<0.001

*Categorical variables were expressed as numbers (%). Continuous variables were expressed as mean ± SD or median (25 and 75% quartile) for non-normally distributed variables*.

*BMI, body mass index; SBP, systolic blood pressure; DBP, diastolic blood pressure; HbA1c, glycosylated hemoglobin; FPG, fasting plasma glucose; TC, total cholesterol; TG, triglyceride; HDL-C, high-density lipoprotein cholesterol; eGFR, estimated glomerular filtration rate; LDL-C, low-density lipoprotein cholesterol; CRP, C-reactive protein; ESR: erythrocyte sedimentation rate; ALT, alanine aminotransferase; AST, aspartate aminotransferase; GGT, γ-glutamyltranspeptidase*.

### Risk Factors Between Abnormal VPT and Clinical Variables

Next, we investigated the risk factors of abnormal VPT with a cluster of anthropometric parameters and biochemical indices (see Table 2). The univariate logistic regression analysis indicated a significant association between abnormal VPT and age [odds ratio (OR) = 1.087; 95% CI, 1.061 to 1.113], duration of gout (OR = 1.077; 95% CI, 1.037–1.117), SBP (OR = 1.035; 95% CI, 1.016–1.054), C-reactive protein (OR = 1.014; 95% CI, 1.001–1.027), ESR (OR = 1.018; 95% CI, 1.006–1.031), presence of tophi (OR = 3.445; 95% CI, 1.856–6.395), and eGFR (OR = 1.014; 95% CI, 0.951–0.981) in all the subjects. No significant association was detected between abnormal VPT and BMI, fasting plasma glucose (FPG), urate, and glycosylated hemoglobin (HbA1c) (see Table 2).

**Table 2:** The univariate binary logistic regression analysis to determine risk factors for peripheral neuropathy (VPT > 15 V) in patients with gout.

**Variables**	**Odds ratio**	**95%CI**	***P***
Age	1.087	1.061–1.113	<0.001
BMI	0.997	0.951–1.044	0.885
Height	0.995	0.966–1.025	0.751
Waist circumference	1.026	0.995–1.058	0.096
Duration of gout	1.077	1.037–1.117	<0.001
SBP	1.035	1.016–1.054	<0.001
DBP	1.017	0.990–1.044	0.216
HbA1c	1.463	0.816–2.623	0.201
FPG	1.113	0.679–1.826	0.670
Urate	1.001	0.998–1.004	0.611
TC	0.840	0.590–1.196	0.333
TG	0.885	0.695–1.127	0.322
LDL-c	0.954	0.638–1.427	0.819
HDL-c	1.446	0.355–5.887	0.607
eGFR	0.966	0.951–0.981	<0.001
CRP	1.014	1.001–1.027	0.040
ESR	1.018	1.006–1.031	0.003
Presence of tophi	3.445	1.856–6.395	<0.001

*BMI, body mass index; SBP, systolic blood pressure; DBP, diastolic blood pressure; HbA1c, glycosylated hemoglobin; FPG, fasting plasma glucose; TC, total cholesterol; TG: triglyceride; LDL-C, low-density lipoprotein cholesterol; HDL-C, high-density lipoprotein cholesterol; eGFR, estimated glomerular filtration rate; CRP, C-reactive protein; ESR, erythrocyte sedimentation rate*.

Subsequently, variables included in the final multivariate logistic regression model were selected according to their clinical relevance and statistical significance in the univariate logistic regression analysis (cutoff: *P* = 0.05) (see Table 3). An elevated age and presence of tophi were associated with a higher risk of abnormal VPT in patients with gout, with an OR of 1.094 (95% CI, 1.061–1.128, *P* < 0.001) and 1.048 (95% CI, 1.002–1.096, *P* = 0.042).

**Table 3:** The multivariate binary logistic regression analysis of risk factors for peripheral neuropathy (VPT > 15 V) in patients with gout.

	**Odds ratio**	**95%CI**	***P***
Age	1.094	1.061–1.128	<0.001
Presence of tophi	1.048	1.002–1.096	0.042

## Discussion

This study focuses on the prevalence and risk factors of PN (assessment by abnormal VPT) in patients with gout. We assessed the association between the VPT levels and various clinical factors and our results suggested that the VPT level was positively correlated with age, duration of gout, SBP, C-reactive protein, ESR, and presence of tophi and negatively correlated with eGFR in all the subjects. Our major finding of this study was that age and presence of tophi were independently associated with a higher risk of PN (assessment by abnormal VPT) in patients with gout.

The prevalence of PN in gout was 11.1% in this study (mean age, 45.5 years; mean gout duration, 7.4 years). There is currently a lack of available information on the prevalence of PN in gout. A previous study reported (included 162 patients with gout) that patients with gout had a frequency of PN of 65.4% (mean age, 49.4 years; mean gout duration, 14 years), which according to questionnaires and nerve conduction studies (NCSs) to assess the presence of neuropathy (17). This large variation of prevalence of PN may be attributed to individual differences, race, discrepancies in gout duration, different study designs, and differences in the diagnostic criteria employed. Another important reason is that 72% of patients have tophi in the above study, while the prevalence of tophi in patients with gout was only 26.92% in this study.

Vibration sensing threshold technology has been widely used for the early detection of PN. VPT assesses the influence of peripheral nerves in a quantifiable manner. The correlation between VPT and DPN has been proved by numerous studies (11, 18). In addition, elevated VPT has been shown to be a dangerous sign for future foot ulcers (19). However, up to now, no study specifically investigated associated factors with VPT in patients with gout. A variety of factors are related to VPT and the correlation between age and VPT has been widely reported, both in the general population (20) and in patients with diabetes (11). Similarly, this study also confirmed the independent correlation between abnormal VPT and age in patients with gout. Possible reasons include age-related reduction in the receptor density, possible degeneration of corresponding peripheral nerve fibers, and morphological modifications of the remaining receptors (21, 22). Therefore, for older patients, the presence or absence of neuropathy should not be considered solely based on the VPT value, but should be based on more medical history, symptoms, and signs to determine its correlation. In addition, as an arthritic disease, gout itself will have some symptoms and signs, such as numbness, pain, and pins and needle sensation, which are easily confused with the symptoms and signs caused by PN. The complex and extensive clinical presentation of gouty neuropathy makes quantitative diagnosis and screening quite difficult. At present, there are no unified diagnostic criteria for gouty neuropathy. In view of the wide application of VPT in the diagnosis of PN, this study used VPT to estimate the prevalence and related risk factors of gouty neuropathy. Further research is needed in the future to assess the correlation between PN and VPT in patients with gout.

Apart from age, the disease duration is also one of the important related factors. Santos et al. (11) have found a positive association between VPT and disease duration in patients with diabetic. This study also showed a similar result. Previous studies have reported the correlation between height and VPT in non-diabetic subjects (20) and patients with diabetes (11) and no similar correlation was found in our gout population. A significant association was also found between ESR (inflammatory markers of disease activity) and abnormal VPT in this study. Previous research also reported that colchicine, used in the therapy of gout, especially in patients with transplantation, is toxic for peripheral nerves and may lead to an acute myopathy with neuropathy (23). In this study, there was no significant difference in the use of colchicine between the two groups and these patients did not regularly use colchicine for a long time.

Recently, various epidemiological studies had reported the association between serum urate and PN in patients with diabetes mellitus and in healthy subjects. A multicenter cross-sectional study in Thailand concludes that the serum urate level was independently associated with PN in patients with type 2 diabetes mellitus (T2DM), particularly in women (24). Lin et al. (25) also showed a significant association between elevated serum urate levels and DPN. Abraham et al. (8) reported that the serum urate levels were correlated with the clinical and electrophysiological severity of diabetic sensorimotor polyneuropathy. However, another study reported that lower serum urate level correlated with an abnormal VPT, an indicator of large nerve fiber dysfunction, in male patients but not in female patients with T2DM (15). In healthy subjects, Abraham et al. (9) showed that higher urate levels correlate with lower sensory nerve function in healthy subjects, expanding the evidence of possible negative influence of urate on peripheral nerves. However, in this study, no correlation was found between serum urate levels and abnormal VPT in patients with gout. The possible reason might be all our patients had gout and their urate levels were in a relatively narrow range. Prospective follow-up studies or drug intervention studies, lowering urate level to normal, to explore the relationship between urate and VPT, and for improvement in the neuropathy will help to draw better conclusions.

Another important observation from this study was the independent association of tophi with abnormal VPT in patients with gout. Gouty tophi represent a symptom of chronic form of gout resulting from the accumulation of monosodium urate (MSU) crystals in tissues. MSU crystals are deposited not only in joints, but also in soft tissues, such as bursae, tendons, median nerve, intrinsic muscles, and at the base of carpal tunnel (26–28). Chen et al. disclosed occurrences of median nerve compression due to gouty tophi formation (29). The consequences of tophaceous disease on neuropathy function in patients with gout are not well known and the role of urate in the pathogenesis of neuropathy is also not very clear. A toxic effect of urate on axons, interfering with neurofilaments and impeding axoplasmic flow, is a plausible hypothesis, but requires neuropathologic documentation (30). Previous studies have attributed nerve entrapment, drug toxicity, vasculitis, amyloidosis, and autoimmune phenomenon as possible causes of PN in patients with rheumatoid arthritis.

This study had several limitations that must be considered. First, nerve conduction study and/or tissue biopsies were not performed; we used VPT to estimate the prevalence of gouty neuropathy, which may result in missed diagnosis. Second, this cross-sectional study lacks the power to track the alterations of serum urate levels longitudinally during the progression of the diseases, making it difficult to determine the causality of serum urate levels for gouty neuropathy and did not address the cause-effect relationship between VPT and clinical variables. Third, participants recruited from a single hospital might limit the generalizability of study findings.

In summary, the risk for PN was increased with an elevated age and presence of tophi in patients with gout. The prevalence of abnormal VPT in gout was 11.1% in this study. These findings may provide useful information for researchers exploring the pathogenesis of gouty neuropathy and reinforce the importance of early detection and timely intervention of PN in individuals with gout. Further longitudinal study is warranted to confirm these findings and to investigate the benefit of strategies that reduce urate or treatment of tophi to prevent PN in people with gout.

## Data Availability Statement

The original contributions presented in the study are included in the article/supplementary material, further inquiries can be directed to the corresponding author.

## Ethics Statement

The studies involving human participants were reviewed and approved by the Ethics Committee of Shanghai Jiaotong University Affiliated Sixth People's Hospital. The patients/participants provided their written informed consent to participate in this study.

## Author Contributions

KG analyzed the data and wrote the manuscript. NL, MW, and LC analyzed and acquired the data. HC contributed to the study conception and design, critical revision of the manuscript, and approval of the final version of the manuscript. All the authors approved the submitted version of the manuscript.

## Funding

This study was supported by the National Key R&D Program of China, Synthetic Biology Research (Grant Number 2019YFA0904500) and the National Natural Science Foundation of China (Grant Numbers 81670737, 81870616, and 82170904) to HC. This study was also financially supported by the National Natural Science Foundation of China (Grant Number 82000780), the Shanghai Minhang District Municipal Health Committee (2020MZYS05), the Basic Research Program of Minhang Hospital (2019MHJC06), and the Shanghai Municipal Health Commission (20194Y0250) to KG. The funders had not participated in the study design, data collection and analysis, or preparation of the manuscript.

## Conflict of Interest

The authors declare that the research was conducted in the absence of any commercial or financial relationships that could be construed as a potential conflict of interest.

## Publisher's Note

All claims expressed in this article are solely those of the authors and do not necessarily represent those of their affiliated organizations, or those of the publisher, the editors and the reviewers. Any product that may be evaluated in this article, or claim that may be made by its manufacturer, is not guaranteed or endorsed by the publisher.
